# An Ounce of Discretion Is Worth a Pound of Wit — Ergonomics Is a Healthy Choice

**DOI:** 10.1371/journal.pone.0071891

**Published:** 2013-10-18

**Authors:** Rehana Rehman, Rakhshaan Khan, Ambreen Surti, Hira Khan

**Affiliations:** 1 Physiology, Bahria University Medical & Dental College, Karachi, Pakistan; 2 BUM&DC, Karachi, Pakistan; 3 Anatomy, Bahria University Medical & Dental College, Karachi, Pakistan; 4 Elixir Technologies, Islamabad, Pakistan; University of Bath, United Kingdom

## Abstract

**Objective:**

The objective of the study was to identify the occurrence and outcome of low back ache amongst computer users and their relation to age, gender, occupation and duration of computer use.

**Materials and Methods:**

A self reported questionnaire tailored from Occupational Health and Safety Act of the Ministry of Labor, Ontario, Canada was used.

**Results:**

416 participants 55.5% males and 45% females using computers for a minimum of five years with age range 22 to 59 years belonged to different occupational groups. Consecutive hours of computer work was found to be associated with work related backache or discomfort in 27.4% (n = 114) participants (16.1% male, 11.3% female). Frequent short breaks improved backache (p value <0.001) in 93 (22.4%) participants (13.2% male, 9.2% female). No significant relation was observed with the duration of computer usage or usage per day; between the two genders or occupational groups. Backache had no significance within age groups.

**Conclusion:**

Our study identifies the occurrence of low back pain among those who are using computer for consecutive hours without breaks and the results suggest the need to create health awareness especially use of short breaks to minimize the risk and occurrence of low back pain. The result of this study can also be used to improve ergonomic design and standards.

## Introduction

With the advancement in technology, computer usage has gained immense popularity irrespective of gender, age, occupation and rationale of use, entrancing its users to spend unaccounted hours in front of computer screens, thereby increasing the risk of muscle tension, discomfort and consequent multiple musculo -skeletal disorders (MSDs) [1]. The muscular discomfort and repetitive strain injuries in turn lead to long-term disability, difficulties in returning to work [2] and psychological discomforts, mental stress and poor productivity at work organization [3].

World Health Organization defines Low back pain (LBP) as: “It is neither a disease nor a diagnostic entity of any sort. The term refers to pain of variable duration in an area of the anatomy afflicted so often that it is has become a paradigm of responses to external and internal stimuli. Such pain ranks high (often first) as a cause of disability and inability to work, as an interference with the quality of life, and as a reason for medical consultation. In many instances, however, the cause is obscure, and only in a minority of cases does a direct link to some defined organic disease exist.”

Experts predict that one in six of employers will be affected by bad ergonomics in one year alone [4] and reported that computer-related vision ailments and musculoskeletal disorders affect millions of computer users every year. Most occupational illnesses are now attributed to repetitive strain injuries leading to conditions such as back pain, neck pain, tendonitis or some other ergonomic causes [5] ^.^ Back pain is the leading cause of sickness and absence from work. As reported by National institute of safety and health” NIOSH”: Thirty million Americans have lower back pain at any given time. It is the second most common cause of work days missed due to illness [6].

Studies on the ergonomic causes of LBP reveal that poor awkward postures cause fatigue, strain and eventually pain. Poor posture may result in structural deformation of the body, muscular contractures, pain in the back and legs, decreased lung capacity, poor circulation, intravascular pressure, and many irregularities in the body [7]. Prolonged sitting leads to a slackening of the abdominal muscles and curvature of the spine thereby organs of digestion and breathing [8], [9]. LBP is found to be associated with prolonged sitting in uncomfortable (non neutral) postures in majority of computer workers [10].

Proper ergonomic design is thus necessary to prevent repetitive strain injuries, which can develop over time with computer usage and can lead to long-term disability [11], [12]. The objective of our study is to identify the occurrence and outcome of LBP among computer users and to assess if it is related to age, gender, occupation and duration of computer use.

## Materials and Methods

It was an observational cross sectional study conducted from January 2011 - December 2011 after approval from Ethical Review Board of Bahria University. Written informed consent was taken from all participants. A self reported questionnaire on ergonomics was developed using the guidelines of the Occupational Health and Safety Act of the Ministry of Labor, Ontario, Canada; and “Easy Ergonomics for Desktop Computer Users” to assess workstation ergonomics and MSD by identified users [13].

Organizations executing ergonomics in the workplace were identified and included in the study through a formal invitation of participation and explanation of the procedure by description of questionnaire. Participants using computers on a regular basis for a minimum of five years met the inclusion criteria. Those using computers for less than five years or having orthopedic prescription for any MSD were excluded from participation. Based on inclusion/exclusion criteria a list of potential participants (convenient sampling) was obtained from each institution and a letter sent to participants for their willingness to be included in the study. Written informed consent was taken from all the participants who were then given a fifteen minute briefing on terms such as Ergonomics and a few others. Objectives of study and the use of instrument were conducted by the researcher followed by a question and answer session to address any queries. Sample size was found to be 341 out a population of 3000 with ***e*** (margin of error) of 5% and ***z*** (confidence interval) of 95%. SPSS software version 15 was used for data entry and analysis. Values were presented as mean ± SD; SE of mean; Chi square test was applied to evaluate results of test; significant with p value <0.05.

## Results

Questionnaire was distributed to 460 participants. Complete response was acquired by 416 participants; 44 incomplete forms were rejected from the study.

### Demographics

416 participants took part in the study, 55.5% were male while 44.5% female; the age range was 22 to 55 years, the mean being 34.82, median 35 and mode 26 years. The standard deviation was 8.07 years. Participants belonged to different occupational and age groups; 19.2% (12% male, 7.2% female) were from information technology (labeled as IT in tables); 20% (12.5% male, 7.5% females) were marketers (labeled as M); 16.3% (9% male, 7.2% females) comprised of bankers (labeled as B); 15.1% (6.7% male, 8.4% female) were doctors (labeled as D); the group of teachers (labeled as T) had 14.2% participants (6.5% male, 7.7% female) while 15.1% were students (labeled as S) (8.7% male, 6.4% females).

### Age Profile of Participants in Various Occupational Groups

The age profile of participants in various occupational groups is given in Table 1.

**Table 1:** Age profile of participants in various occupational groups.

Occupation	Number (%) of participants in various age groups (yrs)			
	15–25	26–35	36–45	46–55
IT	7 (1.7)	38 (9.1)	25 (6)	10 (2.4)
Marketing	14 (3.4)	40 (9.6)	23 (5.5)	6 (1.4)
Bankers	4 (1)	21 (5)	37 (8.9)	6 (1.4)
Doctors	0 (0)	19 (4.6)	26 (6.2)	18 (4.3)
Teachers	0 (0)	2 (0.5)	43 (10)	14 (3.4)
Students	32 (7.7)	31 (7.5)	0 (0)	0 (0)
Total	57 (13.7)	151 (36.3)	154 (37)	54 (13)

### Computer Usage

43.5% participants (26.2% male, 17.3% female) were using computer for 10 or more years, while 56.5% participants (29.3% male, 27.2% female) were using it for 5–9 years. The length and duration of computer usage as well as the consecutive hours worked each day for each group is illustrated in Table 2 and Table 3.

**Table 2:** Computer usage in different age groups.

Use of computers		Number (%) of participants in age groups (yrs)			
		15–25	26–35	36–45	46–55
Duration	5–9 Yrs	55 (13.2)	112 (26.9)	55 (13.2)	13 (3.1)
	10 yrs/>	2 (0.5)	39 (9.3)	99 (23.8)	41 (9.9)
Length of use/day	<5 hrs	18 (4.3)	25 (6.0)	8 (1.9)	3 (0.7)
	5–7 hrs	19 (4.6)	75 (18.0)	65 (15.6)	26 (6.2)
	8 hrs or >	20 (4.8)	51 (12.2)	81 (19.5)	25 (6.0)
Consecutive hrs	1–2 hrs	37 (8.9)	103 (24.7)	125 (30)	36 (8.6)
	3–4 hrs	20 (4.8)	48 (11.5)	27 (6.5)	14 (3.4)
	5 hrs or >	0 (0)	0 (0)	2 (0.5)	4 (1.0)

**Table 3:** Computer usage in different occupational groups.

Use of computers		Number (%) of participants in occupational groups					
		IT^*^	M^**^	B^***^	D^©^	T^¥^	S^∞^
Duration of use	5–9 Yrs	51 (12.3)	64 (15.4)	34 (8.1)	27 (6.5)	8 (1.9)	51 (12.3)
	10 yrs/>	29 (7.0)	19 (4.5)	34 (8.1)	36 (8.6)	51 (12.3)	12 (2.9)
Length of use/day	<5 hrs	10 (2.4)	11 (2.6)	2 (0.5)	8 (1.9)	0 (0)	23 (5.5)
	5–7 hrs	31 (7.4)	44(10.6)	15 (3.6)	36 (8.6)	31 (7.5)	28 (6.7)
	8 hrs or >	39 (9.4)	28 (6.7)	51(12.2)	19 (4.6)	28 (6.7)	12 (2.9)
Consecutive hrs	1–2 hrs	18 (4.3)	15 (3.6)	15 (3.6)	17 (4.1)	16 (3.8)	16 (3.8)
	3–4 hrs	1 (0.2)	3 (0.7)	5 (1.2)	2 (0.5)	3 (0.7)	3 (0.7)
	5 hrs or >	0 (0)	0 (0)	2 (0.5)	2 (0.5)	2 (0.5)	0 (0)

IT^*^ = Information technology, M^**^ = Marketing, B^***^ = Bankers, D^©^ = Doctors, T^¥^ = Teachers and S^∞^ = Students.

### Backache

Work related backache or discomfort (either alone or in combination) was reported by 27.4% of participants (16.1% male, 11.3% female). 77.9% participants (42.1% male, 35.8% female) had chairs with adjustable backrest that can be inclined to 120 degrees while 22.1% had identical chairs that did not allow such adjustments. 43.02% of these (25.5% male, 17.5% female) were adjusting their chairs for health and safety reasons p<.001. 57.45% of all participants knew the importance of inclining the backrest to prevent the occurrence of MSD while only 43% were actually practicing it p<.001. 19.2% participants who had ergonomically specific chairs suffered from backache (p<.015).

Consecutive hours of work on the computer (without any short breaks) was associated with the occurrence of backache/feeling of discomfort p<0.001. 23.3% of the participants developed lower back pain within 1–2 consecutive hours of work while 4.1% developed it after three or more consecutive hours. The occurrence of backache had no statistical significance amongst occupational groups. The occurrence observed to be more in the 26–35 yrs age group was not significant.

15.4% participants who reported backache were using computers for 5–9 yrs as compared to 12% who were using it for 10 or more years. Occurrence of LBP had no significant relation to the duration of computer usage (p = 0.5). Regarding the duration of use each day, 3.6% were using computers for less than five hours each day, 12% were using it for 5–7 hours and 11.8% for eight or more hours each day. Although the occurrence of backache/discomfort is observed to be more in those spending greater time on computer yet this has no statistical significance (p = 0.9). 22.4% participants (13.2% male, 9.1% female) noticed an improvement in discomfort/LBP p<.001 with frequent short breaks, however this was not significant between the two genders, age groups or occupational groups. 20.7% participants (13.2% male, 7.5% female) self prescribed to relieve backache p<.001. 6.3% participants (3.9% male, 2.4% female) needed consultation with a doctor for backache. 3.6% participants visiting the doctor had already used self prescriptions (not significant). 5.5% participants (3.1% male, 2.4% female) who consulted the doctor were prescribed medicines (p<.001) and only 2.6% (1.6% male, 1% female) were recommended exercises p<.001. On the whole, 2.4% of all participants who suffered from work related backache or discomfort either alone or in combination were prescribed medicines or recommended exercises (p<.001). An extra support was also recommended to 1.7% participants (1.3% male, 0.5% female) p<.001. The outcome of back ache was not related to any particular age or occupational group or between the two genders (Tables 4 & 5).

**Table 4:** Backache in various occupational groups.

Backache & its outcomes		Number (%) of participants in occupational groups						Total
		IT^*^	M^**^	B^***^	D^©^	T^¥^	S^∞^	
Backache		19 (4.57)	18 (4.33)	20 (4.8)	19 (4.57)	19 (4.57)	19 (4.57)	114 (27.4)
Occurs in:	1–2 hrs	18 (4.33)	15 (3.61)	15 (3.6)	17 (4.12)	16 (3.84)	16 (3.84)	97 (23.3)
	3 or < hrs	1 (0.24)	3 (0.72)	5 (1.2)	2 (0.5)	3 (0.72)	3 (0.72)	17 (4.12)
Improves with breaks		16 (3.84)	17 (4.12)	14 (3.37)	15 (3.61)	19 (4.57)	12 (2.88)	93 (22.4)
Self prescriptions		13 (3.13)	12 (2.88)	17 (4.12)	14 (3.37)	14 (3.37)	16 (3.84)	86 (20.7)
Doctor’s visit		6 (1.44)	6 (1.44)	2 (0.5)	3 (0.72)	8 (1.92)	1 (0.24)	26 (6.25)
Medicines prescribed		5 (1.2)	5 (1.2)	2 (0.5)	2 (0.5)	8 (1.92)	1 (0.24)	23 (5.53)
Exercises recommended		1 (0.24)	2 (0.5)	1 (0.24)	1 (0.24)	6 (1.44)	1 (0.24)	12 (2.88)
Extra support		0	2 (0.5)	0	1 (0.24)	4 (0.96)	1 (0.24)	8 (1.92)

IT^*^ = Information technology, M^**^ = Marketing, B^***^ = Bankers, D^©^ = Doctors, T^¥^ = Teachers and S^∞^ = Students.

**Table 5:** Backache in various age groups.

Backache & outcomes		Number (%) of individuals in age groups (yrs)				Total
		15–25	26–35	36–45	46–55	
Backache		13 (3.1)	42 (10.1)	44 (10.6)	15 (3.6)	114 (27.4)
Occurs in:	1–2 hrs	11 (2.6)	38 (9.1)	33 (7.9)	15 (3.6)	97 (23.3)
	3 or < hrs	2 (0.48)	4 (1.0)	10 (2.4)	1 (0.2)	17 (4.1)
Improves with breaks		9 (2.2)	33 (7.9)	37 (8.9)	14 (3.4)	93 (22.4)
Self prescriptions		11 (2.6)	31 (7.5)	31 (7.5)	13 (3.1)	86 (20.7)
Doctor’s visit		2 (0.48)	7 (1.7)	9 (2.16)	8 (1.9)	26 (6.25)
Medicines prescribed		2 (0.48)	5 (1.2)	9 (2.16)	7 (1.7)	23 (5.52)
Exercises recommended		1 (0.2)	3 (0.7)	3 (0.7)	5 (1.2)	12 (2.8)
Using an extra support		1 (0.2)	2 (0.48)	2 (0.48)	3 (0.7)	8 (1.9)

## Discussion

Computer Ergonomics; the engineering science is concerned with studies of human-machine interactions and the physical and psychological relationship between computers and the people who use them. Use, dependence and “user” health concerns of computer [14] are due to lack of knowledge among the employees and employers about safety and preventive measures necessary for the occupational wellness.

LBP is the third leading cause of disability in persons under 45 years of age and is the focus of most studies on early identification and prevention of musculoskeletal pain and disabilities [15]. According to research LBP occurs most often between ages 30 and 50, due in part to the aging process but also as a result of sedentary life styles with too little exercise. The prevalence of LBP increases from childhood to adolescence [16] and peaking between ages 35 and 55 [17]. It affects men and women equally, is the commonest cause of work related disability in people less than 45 years of age and the most expensive reason of work related disability in terms of worker’s compensation and medical expenses. Our study also shows that men and women at work were affected equally by LBP.

Studies further suggest that an excessive hour of sitting in an ergonomically in-correct chair is a major cause of LBP in computer users [8]. Although Callaghan and McGill [18] found that static chairs do not provide enough movement to achieve muscular activation levels or relieve the weight loads put on the lower back, studies of people sitting at work indicate that they tend not to use manual adjustments on their chairs [19]. Our study also supports the limited use of manual adjustment of backrest by the participants; 77.9% had ergonomically specific chairs while only 43% were adjusting backrest for health and safety reasons.

The amount of time (consecutive hours of sitting without a break) especially in the presence of non-ergonomic compliant furniture and/or in-correct sitting posture adds to repetitive LBP. Research conducted by Hakala et al [20] showed a potential dose – response relationship between daily computer usage time and MSDs. Our study also shows a positive relationship between consecutive hours of computer work and the occurrence of lower back symptoms.

Intensive computer work diminishes opportunities to change postures and move. Both, more frequent postural changes and more frequent periods of relaxation of parts of the extensor musculature have been indicated to prevent back discomfort during prolonged sitting [21] defined micro breaks as “scheduled rest breaks taken to prevent the onset or progression of cumulative trauma disorders in the computerized workstation environment.” The study showed that micro breaks have been found to have a positive effect on reducing discomfort in neck, upper back and lumbar spine and are most effective when taken at 20 minute intervals [21]. A field study of supplementary rest breaks for data-entry operators showed that a supplementary five-minute break during each hour which otherwise did not contain a break lowered the discomfort levels without reductions in work performance [22]. Another study compared the effects of frequent short rest breaks with stretching in a larger and a smaller work site. The supplementary work rest schedules also minimized the errors made during work [23] thereby addressing the concern of employers. The regular work hours of our participants included two fifteen minute tea breaks and one forty five minute lunch/prayer break. The response of 22.4% participants confirmed that short breaks improved symptoms of LBP.

LBP is among the most common medical problems difficult to treat. To begin on the positive side, the outcome of acute attacks is not that critical; most episodes of backache resolve within a few weeks without residual functional loss [24]. Regarding the prognosis of LBP (without other health concerns), symptoms subside on their own within a month in up to 90% individuals [25]. In our study, participants relieved themselves of the symptoms by either short breaks or self prescriptions and only 6.3% visited a doctor for LBP, of whom, 5.5% were prescribed medicines and 2.8% were advised additional exercises.

It has already been proved that, with correct ergonomic approach and knowledge and frequent short breaks, a lot of musculoskeletal discomforts and problems can be avoided [20]. Purposeful use of appropriate ergonomic knowledge can thus accomplish some very important objectives like reduction of physical ailments and psychological stress factors [26].

In developed countries a culture has matured where health and safety of employees is prioritized whereas in developing countries occupational health and safety have sadly been taken for granted because of the poor awareness of health ergonomics and lack of policies to create a healthy and occupationally safe environment. It is equally important to make the employers realize the fact that setting ergonomic standards at workplaces requires a small initial investment but the payoff in terms of productivity is well worth it.

## Limitations

It was beyond the scope of the study to verify the ergonomic standards that existed at work stations; we had to rely on the verbal statement of participants.

## Conclusions

Our study highlights the need to call attention to modifiable cost effective factors associated with the health and safety of workers. While strategies to improve working conditions are awaited some preventive modes can be used to create ergonomics awareness and minimize the occurrence of MSDs.

## Supporting Information

Letter S1 **Ethical review letter.**
(JPG)
