# Supplementary figures and images for: An Ounce of Discretion Is Worth a Pound of Wit — Ergonomics Is a Healthy Choice
Source: PLoS One. 2013 Oct 18;8(10):e71891. doi: 10.1371/journal.pone.0071891 (PMC3799755; doi:10.1371/journal.pone.0071891)

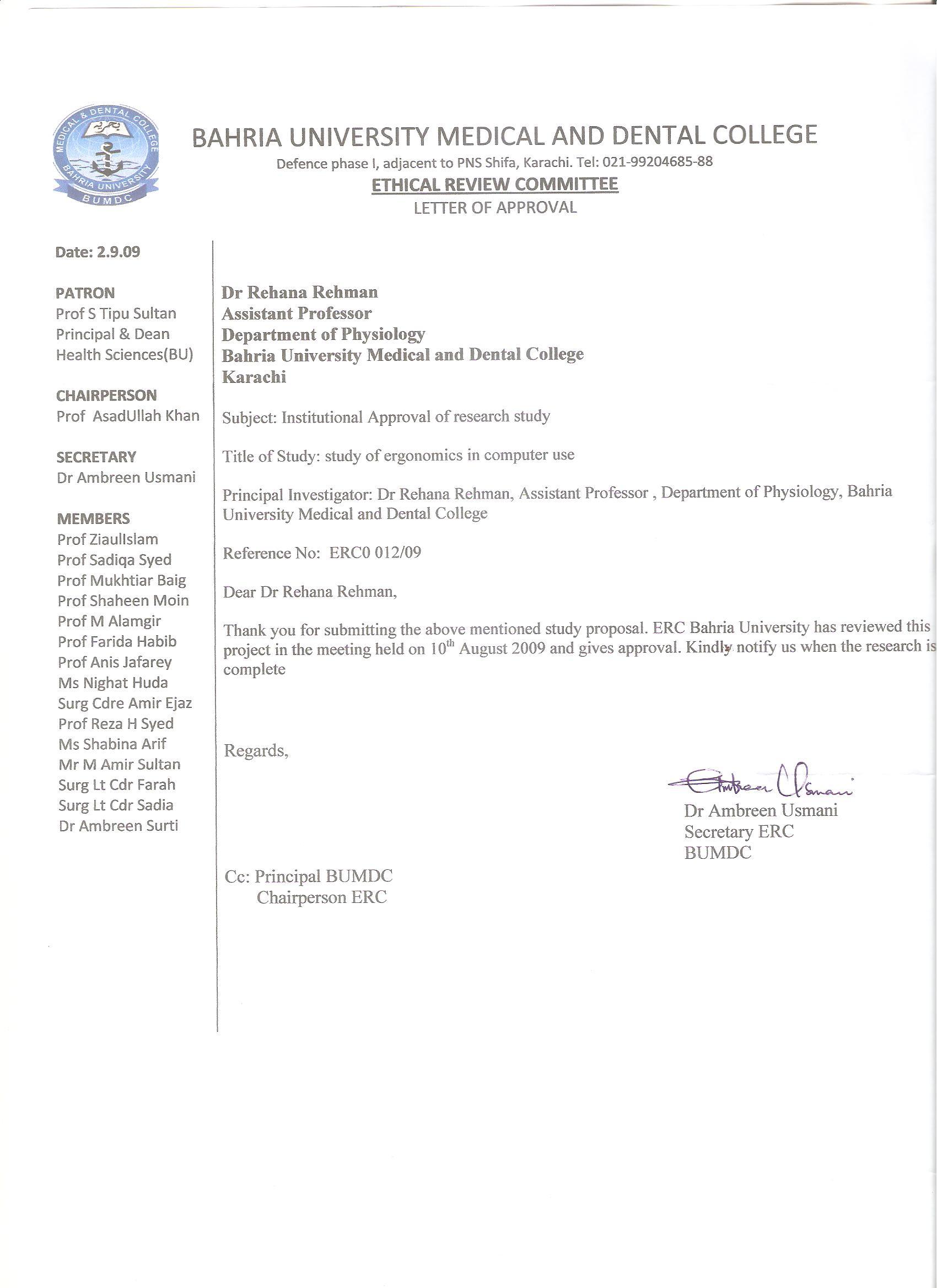

Supplement: Letter S1 — Ethical review letter. (JPG) [file pone.0071891.s001.jpg]
